# Prevention of *Pseudomonas aeruginosa* Biofilm Formation on Soft Contact Lenses by *Allium sativum* Fermented Extract (BGE) and Cannabinol Oil Extract (CBD)

**DOI:** 10.3390/antibiotics8040258

**Published:** 2019-12-10

**Authors:** Valeria Di Onofrio, Renato Gesuele, Angela Maione, Giorgio Liguori, Renato Liguori, Marco Guida, Roberto Nigro, Emilia Galdiero

**Affiliations:** 1 Department of Sciences and Technologies, University of Naples “*Parthenope*”, Business District, Block C4, 80143 Naples, Italy; denevo88@gmail.com; 2 Department of Biology, University of Naples “*Federico II*”, Via Cinthia, 80126 Naples, Italy; renato.gesuele@unina.it (R.G.); angela.maione3@gmail.com (A.M.); marco.guida@unina.it (M.G.); emilia.galdiero@unina.it (E.G.); 3 Department of Movement Sciences and Wellbeing, University of Naples “*Parthenope*”, Via Medina 40, 80133 Naples, Italy; giorgio.liguori@uniparthenope.it; 4 Department of Chemical, Material and Production Engineering, University of Naples “*Federico II*”, Piazzale V. Tecchio 80, 80125 Naples, Italy; roberto.nigro@unina.it

**Keywords:** biofilm, soft contact lens, *Pseudomonas aeruginosa*, *Allium sativum* fermented extract, cannabinol oil extract

## Abstract

Two natural mixtures, *Allium sativum* fermented extract (BGE) and cannabinol oil extract (CBD), were assessed for their ability to inhibit and remove *Pseudomonas aeruginosa* biofilms on soft contact lenses in comparison to a multipurpose Soft Contact Lens-care solution present on the Italian market. *Pseudomonas aeruginosa* (ATCC 9027 strain) and *Pseudomonas aeruginosa* clinical strains isolated from ocular swabs were tested. Quantification of the biofilm was done using the microtiter plate assay and the fractional inhibitory concentration index was calculated. Both forms of *Pseudomonas aeruginosa* generated biofilms. BGE at minimal inhibitory concentration (MIC) showed inhibition percentages higher than 55% for both strains, and CBD inhibited biofilm formation by about 70%. The care solution at MIC inhibited biofilm formation by about 50% for both strains tested. The effect of BGE on the eradication of the microbial biofilm on soft contact lenses at MIC was 45% eradication for *P. aeruginosa* ATCC 9027 and 36% for *P. aeruginosa* clinical strain. For CBD, we observed 24% biofilm eradication for both strains. For the care solution, the eradication MICs were 43% eradication for *P. aeruginosa* ATCC 9027 and 41% for *P. aeruginosa* clinical strain. It was observed that both the test soft contact lenses solution/BGE (fractional inhibitory concentration index: 0.450) and the test soft contact lenses solution/CBD (fractional inhibitory concentration index: 0.153) combinations exhibited synergistic antibiofilm activity against most of the studied bacteria. The study showed that BGE and CBD have good effect on inhibition of biofilm formation and removal of preformed biofilms, which makes them promising agents that could be exploited to develop more effective care solutions.

## 1. Introduction

Diseases related to the eye are frequently observed in clinical practice. Soft Contact lenses have a great impact on improving vision, but their use can often be associated with a risk of infections [1]. Eye infections related to the use of soft contact lenses are linked to various risk factors such as falling asleep with contact lenses, wetting the lenses with water, not replacing soft contact lenses periodically and reusing the disinfectant solution [2].

Several studies have reported that adolescent and young adult soft contact lens wearers present greater risks of contracting eye infections compared to adult or elderly wearers likely because the former have incorrect hygienic practices for maintenance of their soft contact lenses [3,4]. 

The Center for Disease Control and Prevention established that there were about 41 million soft contact lens wearers aged ≥18 years in the United States in 2015, and most of them behaved in a manner that put them at risk of contracting eye infections. In 2016, in the United States, it was estimated that one in seven adolescent and one in six adult soft contact lens wearers stated that they had at least one risky episode of eye infection. They reported falling asleep with soft contact lenses, swimming with soft contact lenses, and replacing the containers and storage solution at intervals longer than recommended [5].

Common ocular pathogens include *Staphylococcus aureus*, *Pseudomonas aeruginosa*, *Escherichia coli*, and other organisms [6]. Previous epidemiological studies identified *Pseudomonas aeruginosa* as the primary causative agent in soft contact lens-related corneal infection [7]. 

The solutions must be able to inhibit the growth of pathogens to protect users from infections [8] and thus to decrease the risk of soft contact lens-related infections [9]. Furthermore, because the formation of bacterial biofilms on soft contact lenses increases infectious eye diseases likelihood, and as biofilms are highly resistant to antibiotics, it is necessary for soft contact lens care solutions to have the ability to reduce or prevent biofilm formation [10,11].

Bacterial cells that colonize a surface within a biofilm show greater resistance to antimicrobial substances than free cells. This phenomenon is attributed to both the lower speed of diffusion of biocides through the biofilm matrix, and the lower levels of oxygen and nutrients that the cells receive compared to the planktonic ones. This results in a lower growth rate, but also less sensitivity to antibiotics and disinfectants [12].

Preventing, reducing or eliminating microbial biofilms from soft contact lenses is now a necessity for improving eye health. Therefore, anti-biofilm coatings and development of anti-biofilm therapies are the most promising goals for reducing the risk of eye infections associated with biofilms [13].

The inherent biofilm resistance to common disinfectants makes the use of natural compounds as “anti-biofilm agents” challenging. Many natural compounds have been used to kill infectious pathogens, and others have been used for eye remedies [14]. Since ancient times, garlic (*Allium sativum*) and onion (*Allium cepa*), have represented important components of typical recipes and traditional healing systems [15]. Mohsenipour and Hassanshahian studied the effects of *Allium sativum* extracts on biofilm formation and activities on six pathogenic bacteria. The abilities of *A. sativum* alcoholic extracts in inhibition of biofilm formation of *S. pneumoniae*, *P. aeruginosa* and *K. pneumoniae* were more than their ability to destroy the biofilm of these bacteria. This study confirmed the ability of garlic extracts to inhibit the attachment of *Staphylococcus* spp., and therefore, their ability to inhibit the biofilm formation of these bacteria. According to the results of this research and other studies performed on extracts and essential oils of *A. sativum*, the antimicrobial potential of this plant was confirmed and the extracts of this plant were shown to be suitable choices against pathogenic microorganisms [16].

Recently, the antibacterial activity of *Cannabis sativa* was also studied. Several researchers noted its activity against various microorganisms and its anti-biofilm ability [17].

To reduce the risk of eye infections associated with biofilms, several studies have been devoted to the development of anti-biofilm coatings and therapies [13].

In the current study, two natural compounds, *Allium sativum* fermented extract (BGE) and cannabinol oil extract (CBD), *Cannabis sativa* metabolite, were assessed for their activity on inhibition and removal of *P. aeruginosa* biofilms on soft contact lenses in comparison to a multi-purpose Soft Contact Lens-care solution found in the Italian market.

## 2. Results

### 2.1. Assessment of Biofilm Formation 

**Figure 1:**
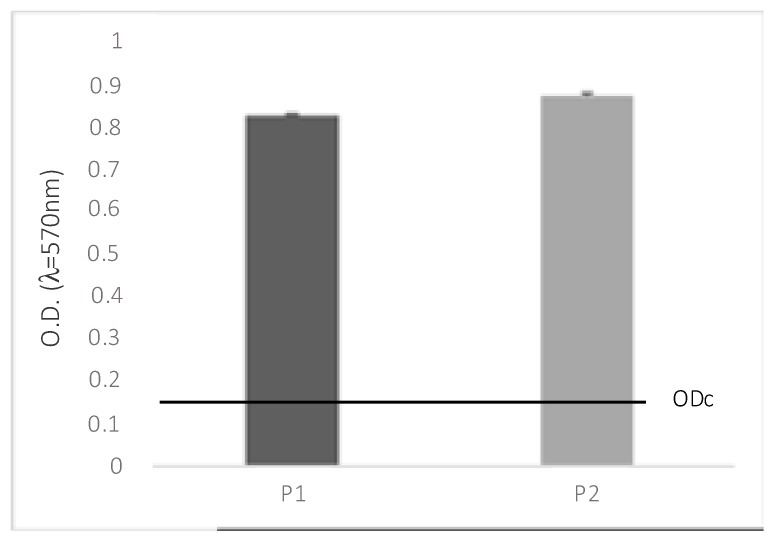
Total biomass of microbial biofilms on soft contact lenses. Negative (OD  ≤  ODc), weak (ODc  ≤  OD  ≤  2.ODc), moderate (2.ODc  <  OD  ≤  4.ODc), and strong biofilm production (4.ODc  <  OD). OD  =  optical density; P1 = *P. aeruginosa* ATCC 9027; P2 = *P. aeruginosa* clinical strain.

Two strains were tested for biofilm production, *P. aeruginosa* (*P. aeruginosa*; ATCC 9027 strain) and *Pseudomonas aeruginosa* clinical strain. Figure 1 shows the total biomass of microbial biofilms on soft contact lenses. The graph highlights that both microbes are capable of forming biofilms on the surface of soft contact lenses and they are classified as strongly biofilm-forming (4.ODc < OD).

### 2.2. Effectiveness of Disinfectant Solution and Natural Compounds on the Inhibition of Biofilms 

The MIC for Soft Contact Lens-care solution was (50%) of the original concentration. While the MIC for BGE and CBD were 20% and 2%, respectively (Table 1).

### 2.3. Prevention of Biofilm Formation

The MIC and sub-MIC of disinfectant solutions were tested for biofilm inhibition capacity. 

**Figure 2:**
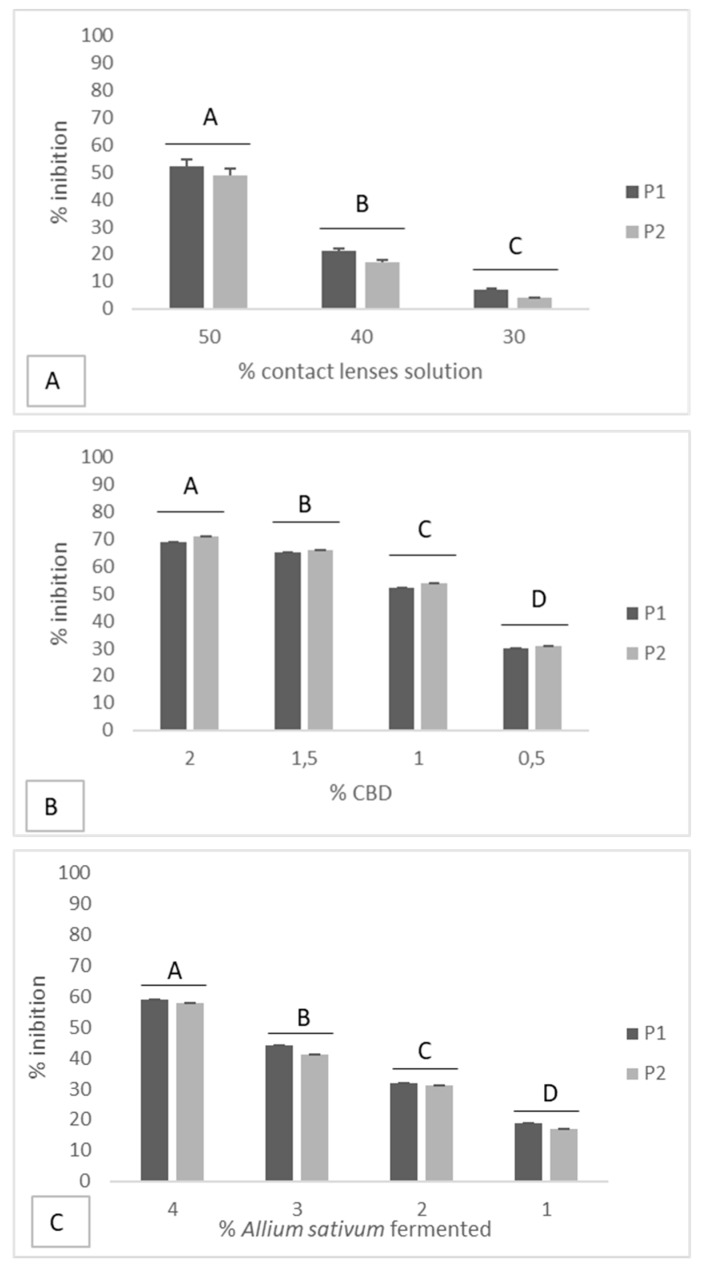
Effect of *Allium sativum* fermented, CBD and Soft CL-care solution on the inhibition of microbial biofilms on soft contact lenses; data with different letters (A–D) are significantly different (*p* < 0.05, T- Test). P1 = *P. aeruginosa* ATCC 9027; P2 = *P. aeruginosa* clinical strain.

For both *Allium sativum* fermented and CBD, $\frac{3}{4}$, $\frac{1}{2}$ and $\frac{1}{4}$ MIC were tested on biofilm-forming *Pseudomonas aeruginosa* (*P. aeruginosa*; ATCC 9027 strain) and *P. aeruginosa* clinical strains, while for the care solution, $\frac{4}{5}$ (40%) and $\frac{3}{5}$ (30%) MIC were tested (Figure 2).

*Allium sativum* extracts at a concentration of 20% (MIC) showed an inhibition percentage higher than 55%, while at a concentration of 10% ($\frac{1}{2}$ MIC), they inhibited biofilm formation by about 35%, for both strains tested.

CBD at a concentration of 2% (MIC) inhibited biofilm formation by about 70%, while at a concentration of 1% ($\frac{1}{2}$ MIC), it inhibited biofilm formation by about 50%, for both strains tested.

Soft Contact Lens-care solution at a concentration of $\frac{1}{2}$ (50%) of the original concentration inhibited biofilm formation by about 50%, while at a concentration of $\frac{4}{5}$ (40%) and $\frac{3}{5}$ (30%) MIC, it inhibited biofilm formation by about 20% and less than 10%, respectively, for both strains tested.

### 2.4. Eradication of Biofilm Formation

Different concentrations of each compound were tested for their biofilm removal effect.

**Figure 3:**
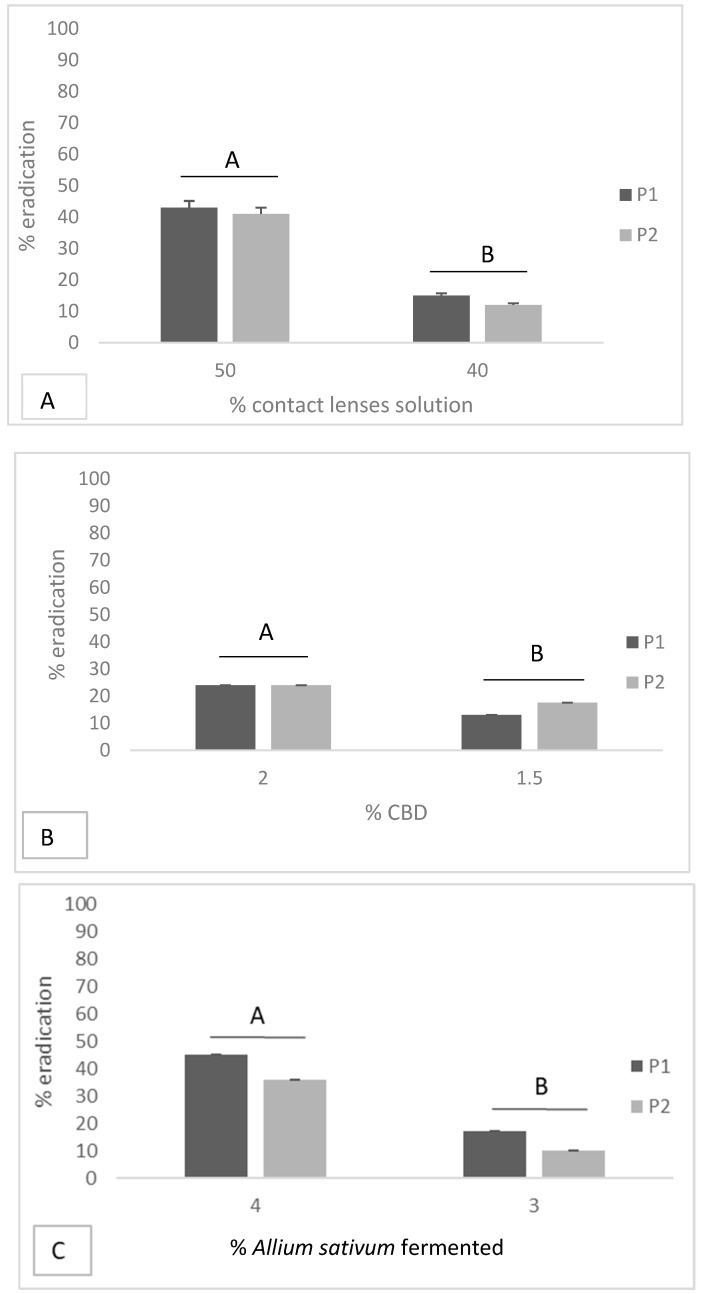
Effect of *Allium sativum* fermented, CBD and Soft CL-care solution on the eradication of microbial biofilms on soft contact lenses; data with different letters (A–B) are significantly different (*p* < 0.05, T-Test). P1 = *P. aeruginosa* ATCC 9027; P2 = *P. aeruginosa* clinical strain.

The effect of *Allium sativum* fermented on the eradication of microbial biofilms on soft contact lenses at MIC was 45% eradication for *P. aeruginosa* ATCC 9027 and 36% for *P. aeruginosa* clinical strain. At a concentration of $\frac{3}{4}$ MIC, it was 17% eradication for *P. aeruginosa* ATCC 9027 and 10% for *P. aeruginosa* clinical strain (Figure 3).

For CBD, 24% eradication of biofilm formed by both strains was observed at MIC, while at a concentration of $\frac{3}{4}$ MIC, there was 13% eradication for *P. aeruginosa* ATCC 9027 and 18% for *P. aeruginosa* clinical strain (Figure 3).

The effect of care solution on the eradication of microbial biofilms on soft contact lenses at MIC concentration was 43% eradication for *P. aeruginosa* ATCC 9027 and 41% for *P. aeruginosa* clinical strain. At a concentration of 4/5 MIC, it was 15% eradication for *P. aeruginosa* ATCC 9027 and 12% for *P. aeruginosa* clinical strain (Figure 3).

### 2.5. Determination of Fractional Inhibitory Concentration Index

Table 2 shows the fractional inhibitory concentration index values of test soft contact lenses solution/BGE and test soft contact lenses solution/CBD combinations on biofilms formed by the studied bacteria. It was observed that both test soft contact lenses solution/BGE (fractional inhibitory concentration index: 0.450) and test soft contact lenses solution/CBD (fractional inhibitory concentration index: 0.153) combinations exhibited synergistic antibiofilm activity against the two strains studied.

## 3. Discussion

Soft contact lens wearers are exposed to an increased risk of developing eye infections on a daily basis, especially when their lenses are not cleaned properly. Soft contact lenses, particularly the soft variety, can provide the ideal reproduction conditions for different pathogens; thus, it is essential that disinfectant solutions are effective against contaminating pathogens to ensure the health of the patient’s eyes [18].

In vivo and in vitro studies have suggested that persistent microbial contaminations of soft contact lenses may be associated with biofilm formation and microbial resistance. A bacterial biofilm can be defined as a structured community of bacterial cells [19]. It is possible that during lens insertion and removal, bacteria may be transferred into the lens storage cases via fingers.

Bacterial biofilms forming in soft contact lens storage cases has been well documented [20].

Although soft contact lens multipurpose solutions meet the international ISO 14729 and FDA 510(k) standard for adequate antimicrobial efficacy, they are only subjected to assessment against selected reference strains of planktonic bacteria and fungi. Antimicrobial activity against attenuated laboratory strains does not ensure efficacy against clinical strains. In addition, commercially available disinfecting solutions may be ineffective against biofilms [21].

The effectiveness of disinfection systems was experimentally tested by Wilson and collaborators. The most effective system for biofilm prevention was 3% hydrogen peroxide. The chlorhexidine base systems were shown to be less effective than peroxide but more effective than some quaternary ammonium derivatives or polyamine polypropylene biguanide [20].

In another study, the effectiveness of disinfectants was tested on the prevention of biofilm formation for long and continuous storage times (6 weeks). The results showed that the contamination rate on the walls of the container is 40% if the storage takes place in solution with polyhexamethylene biguanide, 45% if storage is solution with polyquad, 0% with hydrogen peroxide storage, and 3% if neutralized with a metal catalyst [22].

However, in other studies, the use of hydrogen peroxide was instead associated with a higher degree of container contamination [23,24].

The current study aimed to screen for the biofilm forming from *P. aeruginosa* (*P. aeruginosa*; ATCC 9027 strain) and *P. aeruginosa* clinical strain, and to evaluate the anti-biofilm activity of some natural compounds in comparison to CL-care solution.

Our results showed that the disinfectant solution on the market has moderate activity in inhibiting biofilm formation at MIC concentration (about 50%) without the rubbing step (recommended by the manufacturer but not complied with by some consumers). At the considered sub-minimal inhibitory concentrations, the percentage of inhibition dropped drastically, never exceeding 21%.

We also evaluated the ability of the solution to eradicate the biofilm. The results show a percentage of about 40% for both strains with MIC, but already a percentage lower than 15% at the first tested sub-MIC.

On the contrary, a previous study attributed great efficacy against planktonic bacterial growth to all the solutions tested for the care of soft contact lenses, but a poor activity against bacterial biofilms in vitro [19]. 

The tested organisms were also exposed to minimal inhibitory concentrations and sub-minimal inhibitory concentrations of two natural compounds to evaluate their ability to inhibit and remove biofilms: *Allium sativum* fermented extract and cannabinol oil extract, *Cannabis sativa* metabolite.

Several studies showed the antimicrobial activity of garlic [16,25] and of *Cannabis sativa* [26].

CBD showed the highest activity in inhibiting biofilm formation. Inhibition rates were above 50%, even at sub-minimal inhibitory concentrations.

Instead, *Allium sativum* fermented extract showed higher eradication rates to MICs, while the results are superimposable for sub-minimal inhibitory concentrations.

In the ophthalmological field, *Allium sativum* extracts seem to give good results in solving eye problems and are well tolerated by the eye [27]. 

There has been a resurgence in interest and use of the cannabis plant for medical purposes. The use of cannabis for therapeutic purposes was increasingly limited up to the prohibition of its use with the Single Convention on Narcotic Drugs of 1961. Only several decades later, cannabis has been readmitted as a pharmacological active drug and “medical cannabis” is used and legalized for therapeutic purposes in many countries [28].

Different possibilities of medical use of cannabis have been reported in the literature and, among these, are antitumor effects and treatment of glaucoma [29].

Recently, synergistic combinations of antimicrobials have been proposed to eradicate infections due to multi-resistant pathogens. Some studies indicate the choice of synergistic combination therapy as a preferential treatment in biofilm-associated infections [30]. To evaluate the type of antibiofilm interactions (synergistic, additive or antagonistic), fractional inhibitory concentration index values of test antimicrobials were determined. From the fractional inhibitory concentration index values (Table 2), it was observed that the test soft contact lenses solution/BGE combination showed synergistic antibiofilm efficacy against 69% of test bacterial isolates, whereas this value for test soft contact lenses solution/CBD combination was 75% (data not shown).

Possible mechanisms behind the synergistic interactions of test soft contact lenses solution in combination with BGE and CBD is not clear right now. Studies have shown that two different anti-biofilm mechanisms are able to modulate biofilm formation: inhibition of bacterial surface attachment and interruption of quorum sensing [31]. 

Such scientific evidence led us to test these two natural compounds against biofilms.

## 4. Materials and Methods

### 4.1. Bacterial Culture

*Pseudomonas aeruginosa* (*P. aeruginosa*; ATCC 9027) and *Pseudomonas aeruginosa* clinical strain isolated from ocular swabs were maintained in glycerol stock cultures at −80 °C prior to use and cultured onto Tryptone soy agar (TSA) (Becton Dickinson and Company). Single colonies of bacteria from the overnight cultures were inoculated into tryptone soy broth (TSB) (Becton Dickinson and Company) and incubated in a shaking incubator at 37 °C.

### 4.2. Screened Compounds

One care solution available in the Italian market was tested. It is a sterile isotonic solution, containing polyhexamethylene biguanide at 0.00005% as a preservative active ingredient. Two natural compounds were also tested: BGE (stock solution 175 mg/mL) and CBD (stock solution 3%), *Cannabis sativa* metabolite.

BGE was prepared in the Food Engineering Lab of the Department of Chemical, Material and Production Engineering, University of Naples Federico II. Fresh garlic, bought locally, was fermented for 7 days at a high temperature and high relative humidity (90 °C and RH 70%). The fermented garlic was then pulverized and mixed with distilled water in a 1:1 ratio. Subsequently the aqueous fraction of this mix, the BGE, was separated by a patented extraction process using gaseous norflurane in subcritical condition as a solvent [32]. Cannabinol oil extract (CBD) was purchased from Enecta B.V. (Amsterdam, Holland) (300 mg, 3% CBD).

### 4.3. Biofilm Production

Overnight cultures of isolates from TSA were inoculated in 5 mL TSB and incubated for 24 h at 37 °C. The suspension was diluted 1:100 in TSB to obtain a density of 10^6^ cells/mL. Then, 100 μL of the suspension was added into individual wells, containing silicone hydrogel contact lens (Soft15 energy by Salmoiraghi and Viganò), of polystyrene 24-well plates and incubated at 37 °C for 24 h to allow the develop of the biofilm; media alone was the negative control included. 

The total biomass of the biofilm was analyzed using the crystal violet (CV) staining method [33], as described elsewhere [34]. The content of each well was aspirated and then washed three times with phosphate buffered saline to remove any non-adherent bacteria. The soft contact lens were placed in new 24-well plates at 44 °C for 60 min to allow fixation. 

Then, 150 μL of CV (0.2% p/v) was added to each well and incubated for 15 min. After washing the wells with deionized water, excess stain was gently rinsed off by tap water. Crystal violet bound to the biofilm was detached using 150 μL of 30% v/v acetic acid for 30 min at room temperature, and the absorbance at 570 nm was detected with a spectrophotometer (DR5000, HACH). The test was done in duplicates. Based on the measured optical density, *Pseudomonas aeruginosa* (*P. aeruginosa*; ATCC 9027) and *Pseudomonas aeruginosa* clinical strain were classified into four categories; non-adherent, weakly adherent, moderately adherent, and strongly adherent [35].

### 4.4. Determination of Minimum Inhibitory Concentration of Screened Compounds

Minimum inhibitory concentrations of screened compounds were determined with a microbroth dilution technique as described by the Clinical and Laboratory Standards Institute (CLSI, 2006 M7-A6) with some modifications, using tryptone soy broth [36].

Two fold serial dilutions of each disinfectant agent were prepared using microtiter plates; 100 μL of each dilution were placed in adjacent wells. Then, 100 μL of prepared inoculum was added to each dilution, control wells were included and experiments were made in triplicate. Plates were incubated at 37 °C for 24 h and examined. Wells without the test molecule served as control. The minimum inhibitory concentration was defined as the lowest concentration of compound that completely inhibited visible growth analyzed at 590 nm using a microplate reader (Synergy H4 BioTek) [37].

### 4.5. Effectiveness of Screened Compounds on Inhibition and Eradication of Biofilm Formation

Strains were grown overnight at 37 °C in tryptone soy broth, washed twice in phosphate buffered saline, and suspended to obtain a suspension equivalent to 1 × 10^5^ cells/ml (OD600). Then, 100 μl of each inoculum was dispensed into wells of 24-well microtiter plates.

To prevent cell adherence at the intermediate stage (24 h biofilms) (MBIC), the plates were incubated at 37 °C for 24 h with the soft contact lens solution at 50%, 40% and 30% of its original concentration; with BGE at concentrations of 40 mg/mL, 30 mg/mL, 20 mg/mL, and 10 mg/mL, and with CBD at concentrations of 20 mg/mL (2%), 15 mg/mL (1.5%), 10 mg/mL (1%), and 5 mg/mL (0.5%). 

To eradicate preformed biofilm at the maturation stage (48 h biofilms) (MBEC), the plates were incubated for 48 h, the medium was renewed after 24 h, and disinfectants at the same concentrations were added at the last 24 h. Biofilms formed by bacteria that did not undergo any treatment were used as controls for comparison with the means of the treatments. 

The effect of disinfectants on biofilm inhibition and eradication was quantified by using the XTT assay that analyzed the density of the adhered cells, measuring the relative metabolic activity using the XTT (2,3-bis (2-methoxy-4-nitro-5-sulfo phenyl)-5-(phenylamino) carbonyl)-2H-tetrazolium hydroxide) colorimetric assay kit (Sigma) following manufacturer’s instructions as described elsewhere [38].

Continuous variables were compared using the Student t-test.

### 4.6. Determination of Fractional Inhibitory Concentration Index

The synergistic activity of the test care solution and the two natural compounds was evaluated by calculation of the fractional inhibitory concentration index (FICI) using the method of Ramage et al. [39]. 

Biofilm formation of the test bacterial strains was achieved following the same protocol as described above. After biofilm formation, the medium was aspirated gently and non-adherent cells were removed by washing the biofilms three times with sterile phosphate buffered saline. Then, 100 μL of 2-fold serial dilutions (1/32× minimum inhibitory concentration to 4× minimum inhibitory concentration) of the test care solution and natural compounds were added to each biofilm. The two antimicrobial agents and the test care solution were mixed in the plate crosswise in such a way that the resulting checkerboard contained each combination of the substances in eight doubly increasing concentrations, with wells containing the highest concentration of each substance at opposite corners.

The MBEC of compound combinations, defined as the lowest concentration of substance required to eradicate the biofilm was determined by the XTT reduction assay following the method of Ramage et al. [39].

Fractional inhibitory concentration indices were calculated using the formula: fractional inhibitory concentration index = (minimum biofilm eradication concentration of natural compound in the presence of test care solution/minimum biofilm eradication concentration of natural compound alone) + (minimum biofilm eradication concentration of test care solution in the presence of natural compound/minimum biofilm eradication concentration of test care solution alone). The results were interpreted according to fractional inhibitory concentration indices as follows: ‘synergy’ (fractional inhibitory concentration index ≤ 0.5), ‘additive’ (fractional inhibitory concentration index > 0.5–4) and ‘antagonism’ (fractional inhibitory concentration index > 4) [40]. All the experiments were repeated twice.

## 5. Conclusions

The results of this study are supported by previous studies according to which natural compounds could be used as substances that prevented eye infections, especially those caused by the reckless use of soft contact lenses. The current study is the first to assess the anti-biofilm activity of both BGE and CBD on soft contact lens. It showed that BGE and CBD have an excellent effect on inhibition of biofilm formation and removal of preformed biofilms, which make them promising agents that could be added to new more effective care solutions.

The results provide evidence that the test soft contact lenses solution alone and in combination with BGE and CBD may serve as a potential source for treatment of biofilm-associated soft contact lens, hoping for less negative effects on eye health and less problems related to drug resistance.

## Figures and Tables

**Table 1:** Minimum inhibitory concentration (MIC) of BGE, CBD and soft CL-care solutions against tested stains (S.D. = Standard deviation).

Tested Substances	*P. aeruginosa* ATCC 9027	S.D.	*P. aeruginosa* Clinical Strain	S.D.
**BGE**	4%	0.13	4%	0.38
**CBD**	2%	0.31	>2%	-
**CL-care solutions**	50%	0.16	50%	0.23

**Table 2:** Fractional inhibitory concentration values (FICI) of test soft contact lenses solution in combination with *Allium sativum* fermented extract (BGE) and cannabinol oil extract (CBD) against *Pseudomonas aeruginosa* (*P. aeruginosa*; ATCC 9027) and *Pseudomonas aeruginosa* clinical strain biofilms.

Microorganism.	Combinations					
	Soft Contact Lenses Solution + BGE (CLS+B)			Soft Contact Lenses Solution + CBD (CLCS+C)		
	*FIC*	*FICI*		*FIC*	*FICI*	
*P. aeruginosa* ATCC 9027	0.250 (CLS)	0.450	Synergistic	0.150 (CLCS)	0.153	Synergistic
	0.200 (B)			0.030 (C)		
*P. aeruginosa* clinical strain	0.250 (CLS)	0.450	Synergistic	0.150 (CLCS)	0.153	Synergistic
	0.200 (B)			0.030 (C)		
